# Analgesic efficacy of hydromorphone in American alligators (*Alligator mississippiensis*)

**DOI:** 10.3389/fvets.2025.1520172

**Published:** 2025-02-03

**Authors:** Scott E. Henke, David B. Wester, Cord B. Eversole, Javier O. Huerta, Clayton D. Hilton, Kurt K. Sladky

**Affiliations:** ^1^Caesar Kleberg Wildlife Research Institute, Texas A&M University–Kingsville, Kingsville, TX, United States; ^2^Arthur Temple College of Forestry and Agriculture, Stephen F. Austin State University, Nacogdoches, TX, United States; ^3^Department of Surgical Sciences, School of Veterinary Medicine, University of Wisconsin, Madison, WI, United States

**Keywords:** *Alligator mississippiensis*, American alligator, analgesia, hydromorphone, nociception, von Frey, pain management, reptile

## Abstract

**Background:**

American alligators (*Alligator mississippiensis*) are maintained in zoos, aquaria, and farms for educational, research, and production purposes. The standard of veterinary medical care and welfare for captive reptiles requires managing pain and discomfort under conditions deemed painful in mammals. While analgesic efficacy and pharmacokinetic data for several reptile species are published, data with respect to analgesic efficacy in crocodilians are clearly lacking.

**Objective:**

The objective of this study was to determine the analgesic efficacy of hydromorphone in alligators.

**Methods:**

Female American alligators (*N* = 9; 57 months of age) were exposed to mechanical noxious stimuli at multiple anatomic sites using von Frey filaments ranging in size from 1.65 to 6.65 grams-force, and their behavioral reactions recorded. In order to evaluate analgesic efficacy, hydromorphone (0.5 mg/kg SC) was administered in the axillary region to the same alligators and the mechanical noxious stimuli were repeated and behaviors recorded.

**Results:**

Administration of hydromorphone contributed to a range from 62 to 92% reduced avoidance reactions to mechanical noxious stimuli for two anatomic sites (i.e., naris and lateral mandible, respectively).

**Conclusion:**

Alligators did not appear to experience clinically relevant respiratory depression, hypothermia, or other adverse reactions. Therefore, hydromorphone shows promise as an analgesic option to be administered under painful conditions in American alligators.

## 1 Introduction

The American alligator (*Alligator mississippiensis*) is an iconic reptile species of the southeastern United States (1). American alligators are considered a conservation success story as a result of protective federal legislation, the Endangered Species Act of 1973, which was enacted following sharp population declines due to overhunting and habitat loss during the mid-to late twentieth century (1). Today, American alligators and their eggs are collected commercially and raised on farms for meat, leather, and curios (2). Because of their exceptional conservation history, American alligators offer unique opportunities for educational programs within zoos and aquariums (3).

Due to the popularity of maintaining American alligators in zoo, aquaria, and under commercial managed conditions, veterinary medical care and applied animal welfare practices should be considered standard practice for this iconic reptile species. Routine clinical veterinary medical procedures in all reptiles, including crocodilians, may consist of wound cleaning and repair, blood and tissue sampling for diagnostics, diagnostic imaging, and surgical procedures. While there are some published data with respect to sedation and anesthesia of crocodilians, published clinical analgesic data for crocodilians, particularly American alligators, is lacking (4–6). In early reptile analgesia research, young Nile crocodiles (*Crocodylus niloticus*) developed significantly increased limb withdrawal latencies to a thermal noxious stimulus (i.e., hot plate) for at least 8 h with maximal effect after morphine (0.3 mg/kg and 1.0 mg/kg) was administered intracoelomically (7). In this same crocodile study, pethidine (i.e., meperidine) a short-acting μ-opioid agonist used in human and veterinary medicine, had a similar effect at 1.0 mg/kg intracoelomically, but for less than 3 h (7). However, unlike thermal noxious stimuli used in past crocodilian analgesia research, mechanical noxious stimuli have not been applied for use in crocodilian research even though recent evidence suggests that crocodilians have exquisitely sensitive skin mechanoreceptors in several anatomic areas, referred to as integumentary sensory organs (ISOs) (8). These ISOs were determined to convey a mechanical sensitivity exceeding that of primate fingertips, and were primarily distributed throughout the mandible, maxilla, near eyes and on parts of the fore-and hind limbs (8). Given this mechanosensitivity associated with the head and limbs of crocodilians, it seemed logical to take advantage of these anatomical sites (ISOs) to investigate the efficacy of analgesic drugs before and after exposure to a mechanical noxious stimulus.

Hydromorphone is considered a μ-opioid receptor agonist with some δ-opioid agonist activity, but is primarily considered a semisynthetic ketone derivative of morphine with approximately 10 times the potency (9). Although the exact mechanism of analgesic action is not completely understood, hydromorphone is believed to relieve moderate to severe pain by binding to μ-opioid receptors in the brain and spinal cord (10). While hydromorphone analgesic efficacy has not been evaluated in any crocodilian, it was demonstrated to be provide analgesia in red-eared sliders (*Trachemys scripta elegans*) using the thermal hind limb withdrawal nociception model at 0.5 mg/kg SC for up to 24 h (11). Additionally, hydromorphone plasma concentrations were considered analgesic, when compared to analgesic concentrations in mammals, for 12–24 h after bearded dragons (*Pogona* sp.) and red-eared slider turtles were administered either 0.5 or 1.0 mg/kg but, as with morphine, respiratory depression was a side effect at higher doses (12).

With the lack of published data regarding the use of analgesic drugs in alligators, the objective of this study was to evaluate the mechanical antinociceptive efficacy of hydromorphone in American alligators. We hypothesized that hydromorphone would provide antinociception to a mechanical noxious stimulus using von Frey filaments applied to theoretically mechanosensitive anatomic regions in American alligators.

The Texas A&M University–Kingsville IACUC (Protocol Number 2020-06-22) approved this study. Alligators were used in accordance with the Texas Scientific Collecting Permit No. SPR-0620-085.

## 2 Materials and methods

### 2.1 Alligator collection and captivity

Hatchling alligators were collected from southern Texas, in September 2015 at the Tio and Janell Kleberg Wildlife Research Park of the Caesar Kleberg Wildlife Research Institute on the campus of Texas A&M University–Kingsville. Hatchling alligators were raised in captivity in 6, 3000-L tanks, initially in groups of 5 alligators/tank but the number was reduced to 2 alligators/tank when the alligators obtained a snout-vent length of 60 cm. Alligators were fed Mazuri^®^ crocodilian diet (Mazuri Exotic Animal Nutrition, PO Box 66812, St. Louis, MO, 63166), which provided a complete diet of 45% crude protein (minimum), 9.5% crude fat (minimum), 3% crude fiber (maximum), 12% moisture (maximum), and 13% ash (maximum), and mealworms as a supplement.

### 2.2 Pre-treatment (no drugs)

**Figure 1:**
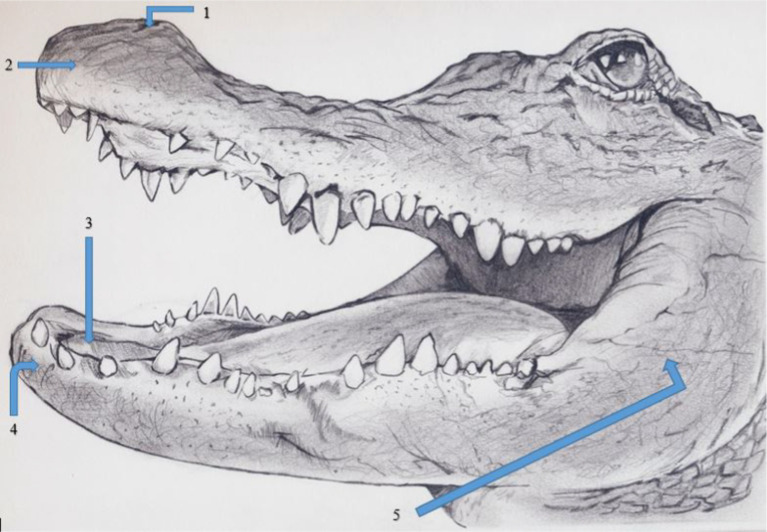
Locations on female American alligators (*N* = 9; *Alligator mississippiensis*) to application of von Frey filaments, with and without hydromorphone, to the (1) naris, (2) center of rostrum, (3) inside of the mouth on the soft tissue behind the teeth of the lower mandible, (4) rostral mandible, and (5) lateral mandible. Figure created by VM Cavazos.

Adult female alligators (*N* = 9, age = 57 months) were manually restrained within their holding tank and weighed (*x̄*= 11.4 kg; range = 9.9–12.7 kg), a temperature sensor was inserted 10 cm into their cloaca, and they were placed in a 90 (L) × 30 (W) × 12 (H) cm restraint cage that allowed freedom of movement for the alligator but provided protection for the researcher. Respiration rate as measured by visual observation of inspiration and expiration, and body temperature were monitored every 30 min. Nineteen von Frey filaments (North Coast Medical, Inc., 780 Jarvis Drive, Suite 100 Morgan Hill, CA 95037 USA) ranging in size from 1.65 to 6.65 grams-force (i.e., 1.65, 2.36, 2.44, 2.83, 3.22, 3.61, 3.84, 4.08, 4.17, 4.31, 4.56, 4.93, 5.07, 5.18, 5.46, 5.88, 6.10, 6.45, and 6.65 grams-force) were applied in progressively larger filament size. Five applications of each von Frey filament were applied to 5 anatomical locations on each alligator: (1) naris, (2) center of rostrum, (3) inside of the mouth on the soft tissue behind the teeth of the lower mandible, (4) rostral mandible, and (5) lateral mandible (Figure 1). These locations were selected because they represent areas on alligators that contain the greatest density of ISOs (8), thus being considered mechanosensitive locations on an alligator’s body. Researchers had easy access to the inside of alligator’s mouths because alligators maintained an open-mouth (i.e., gaping) posture while they were inside the restraint cage. Gaping is a common behavior of alligators during normal respiration and thermoregulation (13). Avoidance reactions to the von Frey filaments were considered positive if alligators responded to the application (i.e., moved, twitched, blinked). We paused 15 s between applications to allow each alligator to relax. The order of applications to anatomic location was randomized; however, once the location was determined, each location received 5 successive applications from each von Frey filament. To determine if alligators were responding to tactile stimuli from the von Frey filaments and not the presence of an approaching researcher, alligators also were blindfolded with a dark black elastic cloth. The elastic cloth was loosely fitted on each alligator so as not to induce a vasovagal response by depressing the eyes (14). Once the blindfold was in place, alligators were allowed 1 min to calm and get accustomed to the blindfold. The order of placing and not placing blindfolds on each alligator was randomized. The number of reactions by each alligator per anatomic location per von Frey filament was recorded and converted into a mean response based on the 5 applications [i.e., percent response method; (15)]. The entire process required 2 h per alligator to complete. This process was repeated with each alligator. These initial behavioral tests were conducted to determine the individual tolerances to noxious stimuli for each alligator.

### 2.3 Treatment (hydromorphone)

Two days following the initial testing, the entire process described above was repeated, after each alligator received a single dose of hydromorphone (0.5 mg/kg SC; West-Ward, Eatontown, NJ 07724, USA) in the axillary region of the right forelimb. Alligators were placed in open-air, covered 1.2 × 1.2 × 2.4 m chain link cages for 45 min to allow the hydromorphone to be absorbed, after which, alligators were placed within the restraint cage as previously described and retested for reactions to the same mechanical noxious stimuli. The time required for hydromorphone absorption was determined prior to this experiment by administering hydromorphone to alligators and recording the time when alligators ceased to respond to a tail grab.

### 2.4 Statistical design and analysis

Because individual alligators can display differing levels of tolerance to provocation (16), alligators served as their own control in a paired design. Each alligator was tested for their reaction to stimuli four times: twice during the pretreatment (i.e., no drug) phase and twice during the treatment (i.e., hydromorphone) phase and each phase was conducted with and without a blindfold. Data were analyzed with a linear mixed model with vision, drug, sensory location, and von Frey filament force, as well as their interactions, as fixed effects; random effects included individual alligator (hereafter, ID); the crossed interaction between ID and vision; the crossed interaction between ID and drug nested in vision; and the crossed interaction between ID and sensory location nested in vision and drug (17–19). This model accounted for the repeated measures nature of our experiment, wherein each animal (a nuisance effect) supported a full combination of all fixed factors. Satterthwaite’s method was used to estimate degrees of freedom (20). Interactions were followed by tests of simple main effects, and when appropriate, simple effects to control type I error rates (18, 21). The response variable (number of positive reactions out of 5 stimuli) is a binomial random variable; therefore, a normal distribution was not expected. Use of a generalized linear mixed model with a logit link failed to converge, and, therefore, the percent response using an angular transformation was analyzed: $Y^{\prime }=\sin^{-1}\left(\sqrt{Y/n}\right)$ , where *Y* = the number of positive reactions and *n* = number of stimuli (21). Effectiveness of hydromorphone in reducing sensory perception also was quantified by comparing area under the curve of the sensory response (percent of reactions to 5 stimuli plotted as a function of von Frey filament for each location and drug condition); areas were computed using the trapezoid rule.

## 3 Results

**Figure 2:**
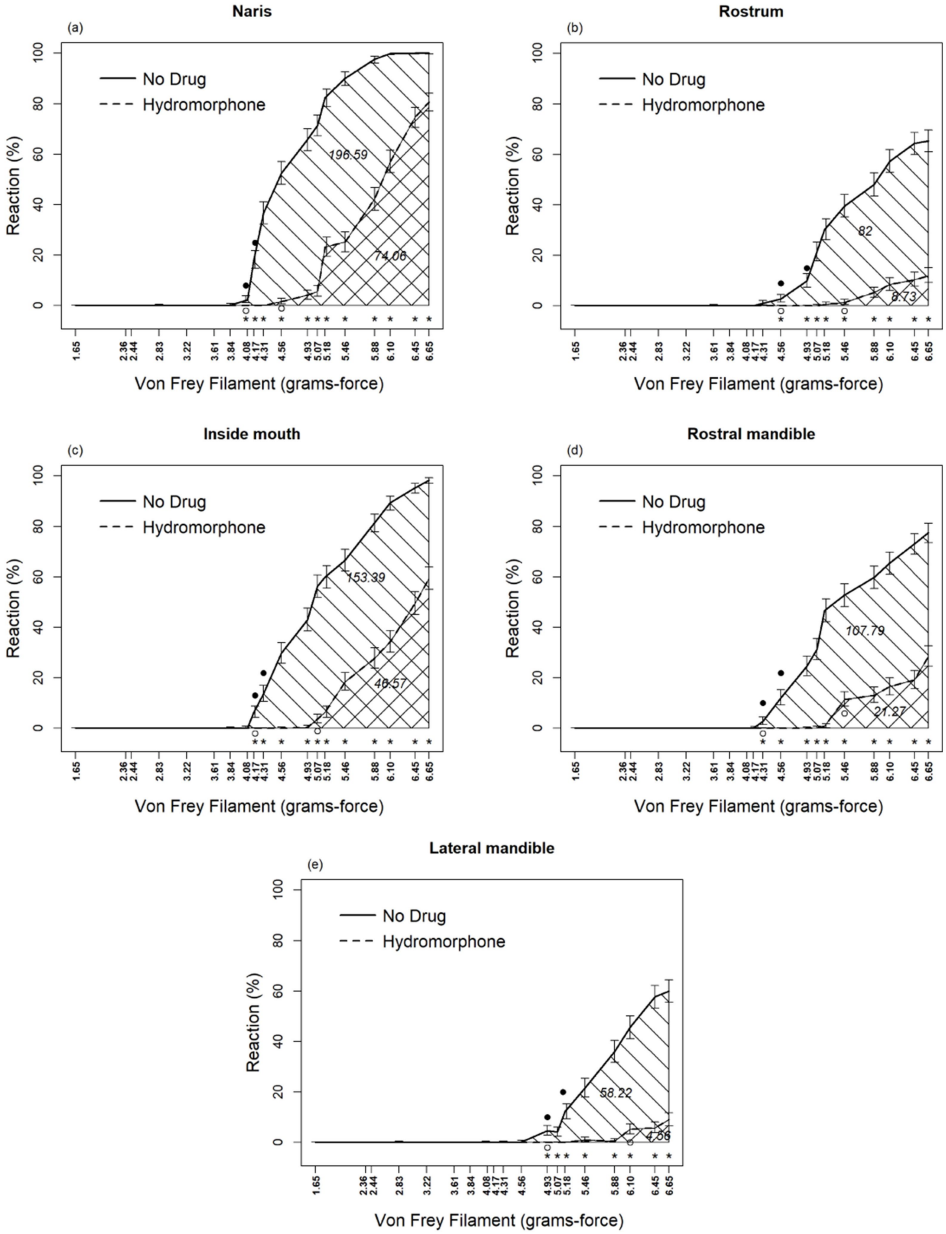
Percent reaction of American alligators (*N* = 9; *Alligator mississippiensis*) to application of von Frey filaments, with and without hydromorphone, within the naris **(A)**, rostrum **(B)**, inside of mouth **(C)**, rostral mandible **(D)**, and lateral mandible **(E)**. * Represents statistically significant differences between treatments by reaction of alligators to different size von Frey filaments. Numbers embedded in cross-hatching are areas under curves; the value under the curve for No Drug, although embedded in the single cross-hatched area, represents the entire area under this curve including both single-and double-cross hatched areas; the value under the curve for Hydromorphone, embedded in the double cross-hatched area, represents only the double cross-hatched portion of the figure.

Hydromorphone lessened the avoidance behavior associated with the application of the von Frey filament mechanosensory stimuli. Drug, sensory location, and von Frey filament diameter interacted (*F*_72, 2,878_ = 4.03, *p* < 0.0001; Table 1) with each other in their effects on alligator sensory perception. Differences in sensory perception by alligators occurred between hydromorphone and no drug at filament sizes ≥4.08, 4.17, 4.31, 4.56, and 4.93 grams-force for naris, rostrum, inside of mouth, rostral mandible, and lateral mandible, respectively (Figures 2A–E). Without hydromorphone, increased sensitivity beyond this force level was experienced when filaments reached 4.17, 4.31, 4.56, 4.93 and 5.18 grams-force for these locations, respectively. With administration of hydromorphone, however, sensitivity beyond this force level was experienced at filaments 4.56, 5.07, 5.46, 5.46, and 6.10 grams-force, respectively. Hydromorphone delayed the response by 3, 4, 4, 5, and 5 filament sizes at the naris, rostrum, inside of mouth, rostral mandible, and lateral mandible, respectively (Figures 2A–E). For example, alligator response in the naris occurred initially with the 4.08 grams-force von Frey filament, but on average, hydromorphone delayed the response until the 4.56 grams-force von Frey filament (i.e., 3 filament sizes larger; Figure 2A).

**Table 1:** Analysis of fixed effects of drug (i.e., no drug versus hydromorphone), vision (i.e., blindfolded versus vision), sensory location (i.e., naris, inside of mouth, rostrum, rostral mandible, and lateral mandible), von Frey filament size (i.e., 1.65, 2.36, 2.44, 2.83, 3.22, 3.61, 3.84, 4.08, 4.17, 4.31, 4.56, 4.93, 5.07, 5.18, 5.46, 5.88, 6.10, 6.45, and 6.65 grams-force), and their respective interactive effects on the reaction of American alligators (*N* = 9; *Alligator mississippiensis*) to the application (*N* = 5) of von Frey filaments.

	Degrees of freedom			
Effect	Numerator	Denominator	*F*	*P* > *F*
Vision	1	24	4.5	0.0444
Drug	1	24	92.4	<0.0001
Drug × Vision	1	24	0.2	0.6469
Location	4	128	171.5	<0.0001
Location × Vision	4	128	2.4	0.0570
Location × Drug	4	128	14.02	<0.0001
Location × Drug × Vision	4	128	0.10	0.9844
Filament	18	2,878	750.9	<0.0001
Vision × Filament	18	2,878	4.3	<0.0001
Drug × Filament	18	2,878	114.6	<0.0001
Drug × Vision × Filament	18	2,878	1.6	0.0497
Location × Filament	72	2,878	22.4	<0.0001
Location × Vision × Filament	72	2,878	0.9	0.6621
Location × Drug × Filament	72	2,878	4.0	<0.0001
Location × Drug × Vision × Filament	72	2,878	0.6	0.9926

**Figure 3:**
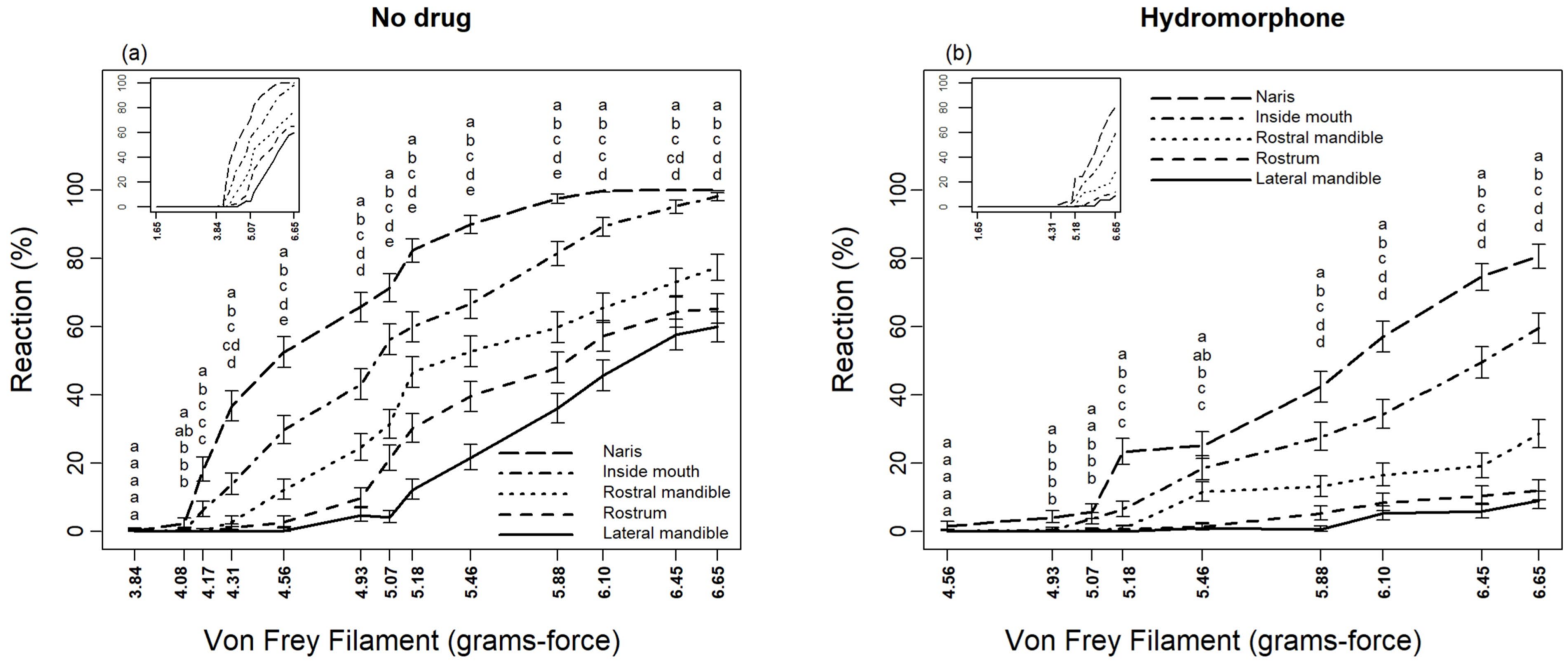
Differing reactions of American alligators (*N* = 9; *Alligator mississippiensis*) to application of von Frey filaments, **(A)** without and **(B)** with hydromorphone, within the naris, rostrum, inside of mouth, rostral mandible, and lateral mandible. Stacked letters above lines are vertically arranged (from top to bottom) in the order of percent reaction from greatest to least (i.e., naris, inside of mouth, rostral mandible, rostrum, and lateral mandible, respectively). Locations for a given filament diameter followed by the same letter are not significantly different (*p* > 0.05, protected LSD test).

Sensory perception differed significantly among locations for alligators without hydromorphone (*F*_4,2,527_ > 2.59, *p* < 0.0352) as well as with hydromorphone (*F*_4,2,527_ > 4.69, *p* < 0.0352), although these differences depended on filament size (Figures 3A,B). Alligators displayed a response to von Frey filaments beginning at 4.08 and 4.95 grams-force without and with hydromorphone, respectively. In general, nares were the most sensitive location, followed by inside the mouth, rostral mandible, rostrum, and lateral mandible (Figures 3A,B). It is worth noting that alligators reacted to the largest von Frey filament (i.e., 6.65 grams-force) at each location >60% of the applications, but that level of response was only observed in the nares after alligators were administered hydromorphone.

Areas under the curve represent sensory perception by alligators (Figures 2A–E), which, without hydromorphone, ranged from 196.6 to 58.2 units for naris to lateral mandible, respectively. However, with hydromorphone, the area under the curve decreased. For example, for nares, the area was 74.1, which means that hydromorphone decreased sensory perception by 62.3%. Similar calculations for lateral mandible, rostrum, rostral mandible, and inside of mouth are 92.2, 89.4, 80.3, and 69.6%, respectively.

**Figure 4:**
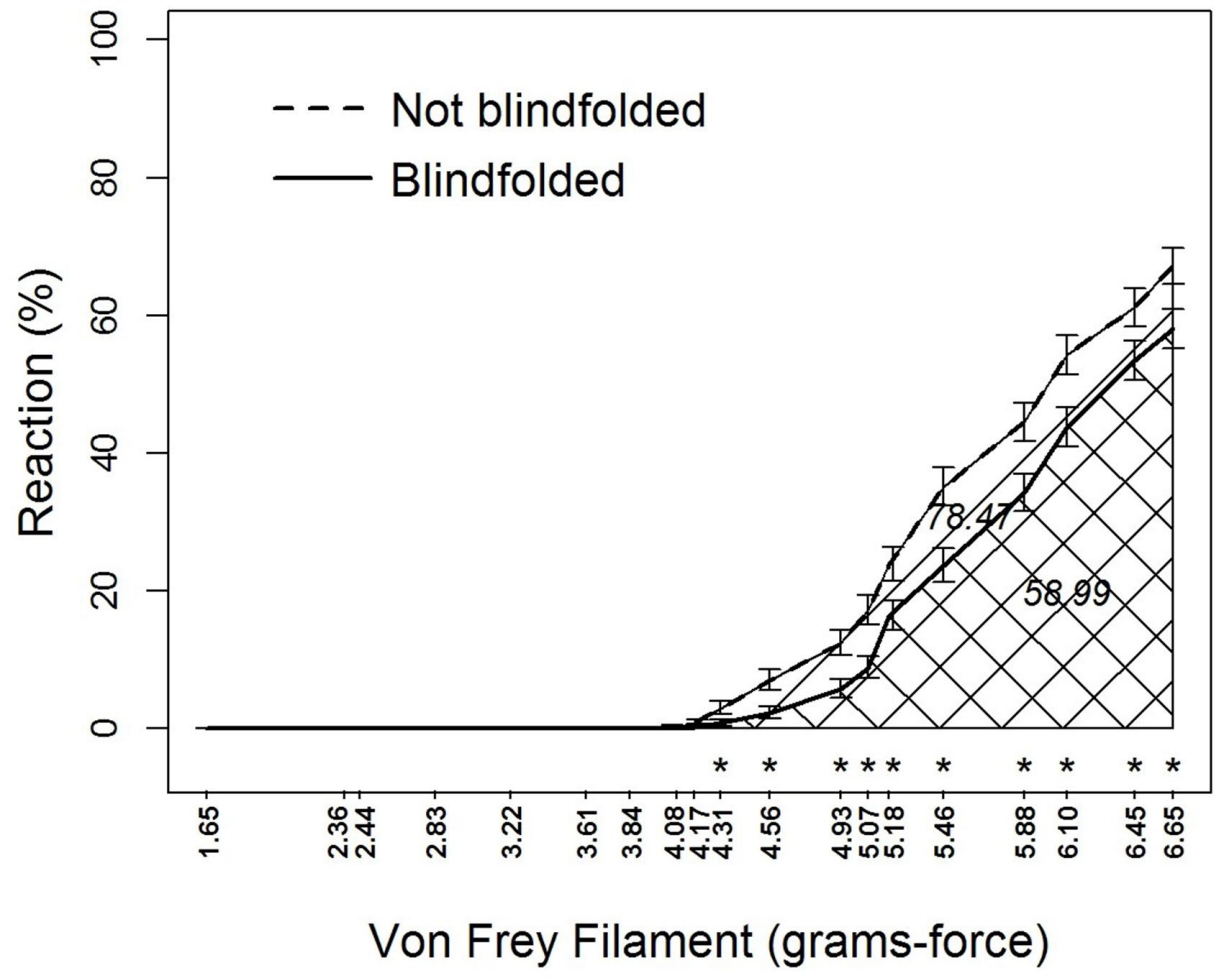
Percent reaction of American alligators (*N* = 9; *Alligator mississippiensis*) to application of von Frey filaments with and without blindfolding. * Represents statistically significant differences between visual and blindfolded alligators by reaction to different size von Frey filaments. Numbers embedded in cross-hatching are areas under curves; the value under the curve for Not Blindfolded, although embedded in the single cross-hatched area, represents the entire area under this curve including both single-and double-cross hatched areas; the value under the curve for Blindfolded, embedded in the double cross-hatched area, represents only the double cross-hatched portion of the figure.

Blindfolding and von Frey filament also interacted (*F*_18,2,878_ = 4.33, *p* < 0.0001) in their effects on sensory perceptions by alligators (Table 1 and Figure 4). Blindfolding reduced sensory perception at filament ≥4.31 grams-force. Area-under-the-curve comparisons showed that blindfolding decreased sensory perception by 24.8%.

No obvious visible signs of sedation, such as lethargy, droopy eyelids, frequent blinking of eyes, unsteady movements, or slower breathing and heart rate, were apparent, nor were adverse reactions to hydromorphone apparent, such as vomiting, lateral shaking of head common in nausea, or excretion. Alligators remained alert and their body temperatures (*x̄* = 31.5 ± 0.8) did not change (*F*_3,256_ = 0.22, *p* = 0.88) with the addition of hydromorphone or fluctuate (*F*_21,256_ = 0.56, *p* = 0.94) during the experiment.

## 4 Discussion

Hydromorphone reduced and delayed the perception of mechanical noxious stimuli in American alligators using von Frey filaments. Hydromorphone inactivates metabolites that have been associated with neuroexcitatory states within the central and peripheral nervous system by acting primarily as a receptor agonist at μ-and δ-opioid receptors to elicit analgesia (22). Morphine (0.8 mg/kg) given intramuscularly has been used on saltwater crocodiles [*Crocodylus porosus*; (7)]. However, morphine has several known adverse side effects, such as respiratory depression and vomiting (10). For example, in red-eared slider turtles, morphine administered subcutaneously caused profound respiratory depression (23). Although hydromorphone is chemically similar to morphine, its structural differences impact the route of metabolism, which may help resolve unwanted side effects (24), albeit the majority of published side effects have been documented in humans (24). In red-eared slider turtles, hydromorphone was antinociceptive using a thermal noxious stimulus model (11). Therefore, use of hydromorphone as an analgesic may be preferable in other reptile species, including crocodilians.

Alligators in managed care are susceptible to traumatic wounds (e.g., bite wounds, lacerations, burns), as well as primary and secondary infections associated with a variety of microorganisms (25). Potential injuries and illness to free-ranging alligators include exposure to toxic chemicals and pesticides (26–28), oral injuries from debris in water (i.e., fish hooks, wooden sticks, etc.), tooth abscesses (29), and skin wounds and lesions from trauma and infection (29). Therefore, the use of analgesic drugs is warranted in alligator management practices and is an important component of standard veterinary medical care for reptiles (30). However, information on the use of analgesics in crocodilians is extremely limited. Published laboratory-based studies in crocodilians demonstrated that morphine, a μ-opioid agonist, attenuates the limb withdrawal behavior after exposure to a thermal noxious stimulus (7). The results of our study support, and build upon these data, such that hydromorphone, also a μ-opioid agonist, diminished the response to a mechanical noxious stimulus in alligators by >60%.

Crocodilians seem to have exquisitely sensitive mechanoreceptors, also referred to as integumentary sensory organs or ISOs, now renamed dome pressure receptors [i.e., DPRs; (31)] on specific anatomical regions of their bodies, particularly the head in alligators and caiman, but are found in nearly every scale of the body surface in other crocodilian species (8). It was hypothesized that these DPRs function as part of a complex mechanosensory system and are adaptive to several ecologically relevant behaviors, including detection of water movements from predators and prey, contact with prey items, and fine oral tactile discrimination (8). These data suggest that mechanoreception may be more salient for crocodilians than thermoreception and the sensitivity of crocodilian DPRs is equivalent to the sensitivity of a primate fingertip, based on the use of very fine diameter von Frey filaments (8). We tested multiple cephalic locations to verify that analgesic effects were centralized and not local effects. While most crocodilians have DPRs on virtually all cranial and postcranial scales, DPRs in alligators are only found on the cranial region (31). Therefore, we tested the analgesic effects of hydromorphone at multiple sites that were deemed sensitive to alligators. Although alligator responses differed by location, hydromorphone delayed and reduced the response to von Frey filaments at each cephalic location. The density of DPRs differs throughout the head of American alligators, which may explain the varying responses observed in this study (32). However, the results of our study differ from that of Leitch and Catania (8), which indicated that stimulation of the nares would elicit a reduced response as compared to stimulation of the rostral mandible.

We recognize that our study has limitations because pain is perceptual and multidimensional and is affected by numerous interactive factors; thus, making accurate measurement difficult at best (33). Genetic strain, age, sex, and reproductive status of animals, stocking density, pen size, and environment can influence the perception of pain (34). Hence, this is the reason why we elected to use same litter, wild-collected eggs, and captive-raised females within same style of pens and similar animal densities until they were nearly 5-years-old. Such confounding variables were experimentally held constant.

There are also limitations to analgesiometry models used in pain research (35, 36). Laboratory-based analgesiometry models cannot completely replicate multidimensional, pathologic pain states. Most of the laboratory-based analgesiometry models rely on the application of thermal (e.g., Hargreaves apparatus), mechanical (e.g., von Frey filaments, toe pinch, hypodermic needle insertion) or chemical (acetic acid test, subcutaneous capsaicin) noxious stimuli and administering a variety of analgesic drugs in order to measure changes (a diminution) in a behavioral, typically a withdrawal, response to the drugs. While these models can certainly cause brief pain or discomfort, they cannot be directly compared to experimental models using surgical or visceral pain. In addition to the experimental model issues, some of the opioid drugs commonly used in pain research, may cause sedation such that a diminution in behavioral responses might be misinterpreted as analgesia, but may realistically be better attributed to sedation.

While the use of Von Frey filaments has never been applied to crocodilians to our knowledge, this method is widely used in mammalian pain/nociceptive research as a mechanical noxious stimulus. We recognize the following limitations associated with using Von Frey filaments in any animal or human subject. Importantly, Von Frey filaments only measure mechanical sensitivity and cannot replicate the multidimensional aspects of pain; in other words, application may not be clinically relevant. When applying the filaments, there is an inherent subjectivity in measuring the response of each subject, such that the observer must interpret the presence of a withdrawal response, which potentially adds variability to the data. For this study, we used one observer in order to decrease interobserver variability and maintain consistency in how the fibers were applied. Data variability also may be associated with application to different anatomical sites on an animal, and the force of filaments can be inconsistent due to variability in application pressure or overuse (damage) to the filaments. The Von Frey filaments used in this study were brand new and the anatomical sites chosen were based on the presence of the most sensitive mechanical nociceptors on crocodilians. Similarly, some individuals may respond more robustly than others due to a variety of methodologic variables (e.g., presence of observer may affect the subject’s response or there may be a lack of subjects’ being appropriately conditioned to the experimental setup). The alligator subjects in this study were conditioned to the experimental environments and blindfolded in order to decrease the effects associated with observer presence. Finally, if the mechanical noxious threshold exceeds the maximum force of the available filament diameters or, alternatively, falls below the lowest possible force of the smallest diameter fibers, data measurements may be less accurate. Withdrawal responses from the alligators in this study were clear and unambiguous.

As had been stated in past studies, anthropomorphism may not be the best way to assess pain, but it is a starting point (37). Although behavior always cannot be treated as evidence of a noxious experience in all animals, there are cases where the degree of deviation in behavior is equal to the degree of pain (38). Thus, we offer our study as a starting point to assess analgesic efficacy of hydromorphone in crocodilians.

Some results presented here are intuitive. For example, increased reactions by alligators to increasing grams-force of von Frey filaments and to the visual stimuli of an approaching filament was expected. However, it was necessary to incorporate these effects into the experimental design to document the antinociceptive effect of hydromorphone in the study subjects. Use of blindfolds is a common practice during capture and restraint in wildlife management to keep animals tranquil (39–41). Our results with alligators were similar to that observed in green iguanas (*Iguana iguana*), in that blindfolds appeared to have a calming effect on the alligators’ responses as well as minimize the presence of the observer (42).

While we did not use pulmonary plethysmography to accurately measure ventilation in our subjects, alligators in our study did not appear to experience respiratory depression based on observational measuring rate of respiration, nor did the alligators experience hypothermia, both of which can contribute to deleterious consequences after administration of anesthetics and analgesics in reptiles (5). Although we do note that ventilation efficiency cannot be assessed by respiratory rate alone, as it is possible for rates to remain constant even with a significant decrease in tidal volume. However, our data suggest that hydromorphone is a potentially effective analgesic choice with negligible, clinically observable respiratory depression in American alligators.

## Data Availability

The raw data supporting the conclusions of this article will be made available by the authors, without undue reservation.
